# Milestones in Understanding Pulmonary Circulation: From Antiquity to Heart-Lung Machine

**DOI:** 10.3390/diagnostics11020381

**Published:** 2021-02-23

**Authors:** Adam Torbicki

**Affiliations:** Department of Pulmonary Circulation, Thromboembolic Diseases and Cardiology, Center for Postgraduate Medical Education, ECZ-Otwock, 05-400 Otwock, Poland; atorbicki@gmail.com

**Keywords:** pulmonary circulation, history, milestones, pulmonary hypertension, renaissance, autopsy, heart catheterization

## Abstract

Human pulmonary circulation is as full of mysteries and surprises as is the history of attempts to uncover and understand them. The Special Issue of *Diagnostics*, appearing after 2020 immobilized the world, give us an opportunity, space and momentum to remind to our medical community at least the main milestones which mark the progress that was made before our times. This review’s aim is to remind about pioneers and their ideas which now are considered as if they were always with us—which is not the case…

## 1. Introduction

Human pulmonary circulation is full of mysteries and surprises, as is the history of attempts to uncover and understand them. Until the 1990s, only a very restricted group of explorers ventured on research journeys through the labyrinth of the pulmonary vascular bed. This no man’s land between cardiology and pulmonology was largely unexplored compared to systemic circulation.

Over the past two decades, the number of clinicians considering themselves experts in pulmonary circulation rose exponentially. Most of them knew everything about current risk stratification in pulmonary hypertension and therapeutic strategies based on three main pathophysiological pathways. They also looked forward, expecting new pathophysiological pathways to be explored and exploited by future therapies. Few had time to look into the past.

This Special Issue of *Diagnostics*, appearing after 2020 immobilized the world, gives us opportunity, space, and momentum to remind our community of some of the milestones which marked the progress made before our time. It is worthwhile to spend an hour or so to learn about people and their ideas appearing over the last 5000 years. Some of these ideas are considered as if they were always with us—which is not the case.

## 2. Where to Start?

In his History of Cardiology published in 1984, Snellen decided to start his narration in 1628 with the publication by William Harvey of his theory on circulation of blood [1]; however, the history of theories involving role of lungs and its vessels is much longer.

## 3. Galen of Pergamon

It seems so strange that for ages healers, physicians, surgeons, and common people were convinced that blood was an ephemeral humoral element. It was supposed to emerge from the liver only to be fully consumed in the tissues shortly afterwards. While today it makes us smile, this was a generally accepted concept. There were no means to calculate blood flow other than looking at open injuries involving large vessels of the victim, be it on a battlefield or during an animal experiment. Even if bleeding were profuse, it would stop soon with the death of the “model”, which could easily be attributed to the inability of the liver to replace large volumes of lost blood on such a short notice.

This concept prevailed for fourteen centuries due to the enormous authority of a single man, who supported it during his lifetime between 129 and 207 A.D.; it was followed almost blindly for the next fourteen centuries. Claudius Galenus of Pergamon, a Greek physician, wiped out any potential alternative concepts [2,3]. In brief, the liver is the source of blood, which it produces from bile derived from the intestines. The heart is not pumping blood but heats it while it moves from right to left ventricles of the heart through the pores in the intraventricular septum. Once heated, blood mixes with the air coming by the pulmonary veins from the lungs. Then it disappears forever, either evaporating across the skin or being utilised by the tissues [2,3].

However, there were some alternative ideas closer to our current understanding, emerging from much more distant history. Nei Ching, a medical compendium from 2900 BC, informed us that “all blood is in the control of the heart and the blood current flows continuously in a circle and never stops”. Whether this theory had any “clinical” implications on Chinese medicine remains unclear and doubtful [3]. Additionally, in Ancient Egypt, the concept of circulation, or at least the importance of pulsatile blood flow, was appreciated. According to Edwin Smith’s papyrus, dating from 1600 BC but likely translated from the time of Imhotep (3000–2500 BC), Egyptians believed that, similarly to the system of channels (metu) irrigating their land, living organisms were “irrigated” in a similar way. They appreciated the pulsation of the heart and knew about the transmission of those pulsations to the peripheral vessels in human limbs [3]. Their knowledge, however, came only from observation of living subjects. Paradoxically, despite enormous experience in embalming, the theological attitude towards human organs prevailed and anatomic observations were not collected [2].

Interestingly, also Galen was not able to benefit from human autopsy observations as in the period of his activity only animal post-mortem studies were allowed. This was one but not the only source of his mistakes, which had to wait until Vesalius to be challenged. The Renaissance broke the dogmatic stability imposed by both the Muslim religion and the Christian Church in many areas of human activities, including anatomy and physiology.

However, this was not without victims.

## 4. Andreas Vesalius

The Renaissance re-opened human corpses to direct post-mortem inspection. The precision of Leonardo da Vinci’s graphs of the heart and vessels are second to none, but they were unfortunately not known to his contemporary medical community. Interestingly, even Leonardo was so influenced by Galen that his most famous drawings of the heart still showed small pores traversing the ventricular septum [4]. We owe the true rebellion against Galen’s anatomic dogmas to Andreas Vesalius (1514–1564). Carefully doing all the autopsy studies himself, he pointed out many errors in Galen’s descriptions, including the presence of communication between the ventricles through already mentioned “pores”, which actually did not exist [5]. The confusion related to the discrepancies in anatomic findings when compared with Galen’ standards was great. Trying to explain them, Vesalius’ mentor, Jacques duBois, ventured a thesis in which Galen was correct, but the anatomy of man must have changed in the over 12 centuries which passed since his time [2].

## 5. Miguel Serveto

Though anatomical discoveries were clearly inconsistent with the general physiologic concept of Galen, Vesalius never questioned it. The most important physiological breakthrough was made by a Spanish theologue and physician, one of the most independent human minds of the XVIth century, Miguel Serveto [2]. Thanks to his education and knowledge of languages—including Greek—he had access to texts which inspired his activities in many directions. He criticized the political ambitions and influences of the Church, pointed out inconsistencies in Catholic and Calvinistic religious dogmas, and last but not least, revolutionized the understanding of the role of the lungs in the human organism. This last accomplishment was probably due to his 12 years of practice and studies in the city of Vienne in France. He summarized his findings in a philosophical treaty “Christianismi Restitutio”, published in Vienne in 1553 [3,6]. He wrote that vital spirit is composed by blood, air, and fire, which are mixed in the lungs supported with blood by the right ventricle. The right ventricle also provides blood for the left ventricle but not directly through the ventricular septum as it was believed, but after a very sophisticated and long passage through the lungs where it mixes with air and returns to the left ventricle ready to serve as a vital spirit [3]. The large diameter of the pulmonary artery, and the fact that it is blood and not air that flows to the left ventricle from the lungs, indirectly supported this theory [6].

Serveto was not given a chance to promote his concepts of pulmonary circulation. He was pursued for his theological theories by both Christians and Calvinists. He managed to avoid the Christians, who only burned his dummy. Imprisoned by Calvinists after a trial, he was considered to be a heretic and was sentenced to a cruel death. He was burned at the stake in Geneva in the same year, 1553, when his treaty including a theory of pulmonary circulation was published. This tragic episode in the history of mankind has been heavily criticised by elites of that time, and since then, giving the death sentence for new ideas would be considered inhuman, making the path for future researchers less dangerous [3].

## 6. William Harvey

The circle was finally closed by William Harvey (1578–1657) [7] in a modest 79 page book published in 1628, 75 years after Serveto’s “Christianismi Restitutio”. He had many arguments to support his thesis that the blood is circulating and re-used many times to nourish the body [8,9]. One argument was that the volume of blood pumped by the ventricles per minute is far too large to be produced by the liver de novo as well as to disappear immediately due to evaporation or consumption in the tissues. In his classical experiment described in “De Motu Cordis”, he compared the effects of two tourniquets applied to the arm. A tight one eliminated peripheral pulses and resulted in cold pale skin and collapse of the superficial veins distally to the tourniquet. In contrast, if a less tight one was applied, without elimination of palpable pulses, it resulted in venous distention and congestion. While Harvey could demonstrate such phenomena related to peripheral circulation, he could only speculate on the presence of similar circulation through the lungs. He considered the diameter of the pulmonary artery too large to serve only to nourish the lungs. Consequently, he suspected that some microscopic vessels connecting the pulmonary artery and the veins in the lungs must be present, but he did not survive long enough to be reassured of their presence, discovered thanks to the invention of the microscope, by Malpighi. However, before his death, he described a post-mortem experiment to one of his German friends, during which he found that water delivered to the lungs through the pulmonary artery was appearing in the left ventricle [10].

## 7. University of Padua

**Figure 1:**
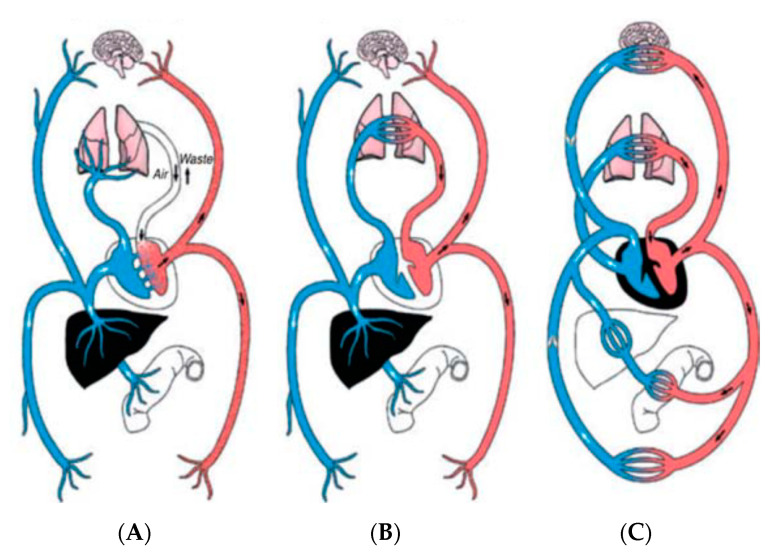
Three concepts of “circulation” by (**A**)—Galenus, (**B**)—Serveto and Columbus, and (**C**)—Harvey. Modified from [3] ElMaghawry M, Zanatta A, Zampieri F. The discovery of pulmonary circulation: From Imhotep to William Harvey, Global Cardiology Science and Practice 2014:31 http://dx.doi.org/10.5339/gcsp.2014.31.

William Harvey and his ideas on circulation of blood are usually assigned to the University of Cambridge where he started his studies. However, Harvey’s medical education was mainly due to the period he spent in Italy under the influence of the most independent group of researchers of the time, affiliated with the University of Padua [7]. Andreas Vesalius (1514–1564) and his anatomical revolt against Galen have already been mentioned. Realdo Colombo (1516–1559), an Italian anatomist and a scholar of Vesalius, suggested pulmonary transit of blood very similar to that proposed by Serveto. They were living and working in the same period, and it is unclear whether their ideas emerged independently or if one was inspired by the other. Fabrizio d’Aquapendente (1537–1619) was the anatomy and surgery professor in Padua by the time William Harvey was studying medicine there. His work suggesting that valves present in the veins determined one-way flow could have been yet another element absorbed from Padua by Harvey, shaping his ideas on the circulation of blood (Figure 1) [3].

## 8. The Overlooked Middle East

While working around 1930 on his doctoral thesis in the Prussian State Library in Berlin, a young Egyptian physician, Muhyo Al-Deen el Tatawi, came across a manuscript written in Arabic and overlooked by western-oriented historians. It was titled “Commentary on Anatomy in Avicenna’s Canon” and written by an eminent Syrian scientist, Ibn-el-Nafis. Tatawi’s thesis was never completed, but it was brought to the attention of Max Meyerhof, a medical orientalist working in Cairo [10]. Meyerhof translated relevant parts of Ibn al-Nafis’ manuscript into several European languages. The manuscript was completed in 1232 by the merely 29-year-old author and appeared more than 300 years before the first European suggestions of the presence of pulmonary circulation. Ibn al-Nafis criticized both Galen and also his great predecessor, Avicenna, for promoting concepts based on false facts, such as the presence of interventricular communication through non-existent pores. The details of the fascinating contribution of Ibn al-Nafis to medicine, comparable to that of William Harvey, as well as the history of the discovery of his manuscript, were described in an article on the Golden Age of Arabic Science by West [10]. In his paper, West also considers the possibility that Renaissance European scientists might have been aware and inspired by Ibn al-Nasif concepts. The Renaissance was a period of great interest in ancient culture and science also coming from outside Europe. However, until the beginning of XXth century, Ibn al-Nafis’ ideas on pulmonary circulation were never cited.

## 9. Early Clinical-Pathological Syndromes

Paradoxically, after the pathophysiological earthquake caused by Harvey, not much happened to our understanding of the pathophysiology of pulmonary circulation for quite a while. Most of what did happen was related to attempts to associate autopsy findings to clinical syndromes. However, some of those observations represented starting points of our current clinical classification of pulmonary hypertension. In 1891, Ernst von Romberg, a German physician, was puzzled by the apparently idiopathic sclerosis of pulmonary arteries, as at autopsy, he was unable to find any disease which could cause or explain it [11]. This is considered by many as the possible first description of “primary” or idiopathic pulmonary hypertension [12]. Soon afterwards, Argentinian clinician Abel Ayerza described a severely cyanotic patient with dyspnoea and polycythaemia, which were also associated with sclerosis of the pulmonary artery at autopsy [13]. Again, today, we would suppose that at least some of the similar cases of “cardiacos negros”, reported also by other authors, represented pulmonary hypertension “secondary” to chronic lung diseases, most likely COPD with a blue bloater phenotype. In the early XIXth century, observations by Morganini and Laenec already linked right ventricular hypertrophy found at autopsy with the presence of chronic lung diseases. However, only the XXth century made it possible to interpret clinical and post-mortem findings with the help of direct hemodynamic measurements.

## 10. Right Heart Catheterization

The introduction of heart catheterisation brought the Nobel prize to three men. From today’s perspective, their selection seems somewhat controversial. This applies particularly to Werner Forssmann, a German physician whose adventurous self-insertion of a large urologic catheter via antecubital vein and his walk downstairs to the X-ray department to check its position within his chest has been widely reported in many different versions. The most likely version of this, and other, Forssman attempts to perform right heart catheterisation and pulmonary angiography, as well as his turbulent life, particularly in the Nazi Germany, is probably best described—despite some risk of bias—in a biographic article written by his daughter [14]. Indeed, Forssmann and his whole family were astounded by the completely unexpected invitation to the Nobel prize ceremony as its recipient. He received it in 1956, 27 years after his first experiment.

Forssmann actually failed to provide clinically relevant measurements or imaging. He was only able to report that intracardiac introduction of a catheter with injection of contrast medium was safe. In contrast, Otto Klein, a Czech physician who, almost immediately after Forssmann, performed a well-designed series of right heart catheterisations, used them to make first-ever reported measurements of cardiac output with Fick’s formula [15,16].

Doctor Klein visited Boston around 1930 and tried to communicate his experience to the eminent cardiological community working there. Stern, in his article dedicated to Otto Kline, mentioned that Andre Cournand remembered this visit. However, it took another 12 years for him and Richards to become interested in RHC and develop it for routine clinical use, for which both of them—together with Forssmann—were awarded the Noble prize [16].

Dr Klein, being of Jewish descent, fled to Argentina in 1939 after Germans entered Prague in 1938. If he had not done so, maybe he would have directly faced Forssmann, who volunteered for Wehrmacht and remained in active service throughout the war [14].

## 11. John Gibbon and His Heart-Lung Machine

What could be a better proof of success in understanding pulmonary circulation if not a man-made machine that effectively replaced the native lungs in its main functions? John Gibbon first thought about such a device as a 26-year-old research fellow at Massachusetts General Hospital in Boston when attending a patient suffering from massive pulmonary embolism [17]. A dramatic attempt to perform a pulmonary embolectomy using the Trendelenburg operation failed, and the patient died. Searching for a solution, Gibbon had an idea to by-pass the pulmonary circulation occluded by thrombi while, at the same time, oxygenating the by-passing blood. This would stabilize the patient and give the surgeon enough time to remove clots from pulmonary arteries. It took him 23 years of continuous effort to move from this idea to its practical implementation. On 6 May 1953, he successfully performed open heart surgery with cardio-pulmonary bypass using a Model II Heart-Lung Machine to close an atrial septal defect. While his heart-lung machine was not predominantly used for its original indication, it initiated unprecedented development in cardiovascular surgery [17]. It also allowed surgeons to perform interventions strictly related to pulmonary circulation, such as pulmonary embolectomy and endarterectomy, correction of complex congenital heart disease involving pulmonary circulation, and finally heart and lung transplantations indicated for intractable pulmonary vascular disease.

## 12. Tribute to Pioneers

**Figure 2:**
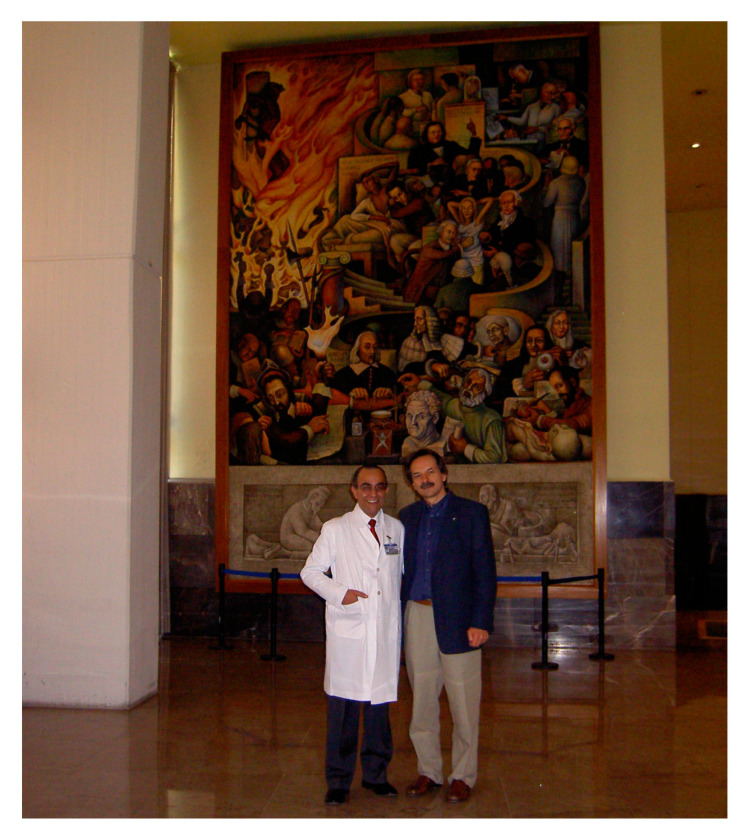
Author with his host, Prof Julio Sandoval, visiting Diego Rivera Mural on History of Cardiological Doctrines at Instituto Nacional de Cardiologia, Mexico DF.

The burning stake is an important symbol depicted on a famous mural painted in 1946 by Diego Rivera for the Instituto Nacional di Cardiologia in Ciudad de Mexico (Figure 2) presenting History of Cardiological Doctrines. Miguel Serveto is painted there just next to William Harvey and together with Galen, Vesalius, Malpighi and many others. When ordering this mural, the director of the Institute, professor Ignacio Chavez, asked Rivera to try to catch the ascending trend of knowledge but also to express how slow and difficult the advance has been. “…how each of those men had to fight routine, prejudice, ignorance and fanaticism [...] If you could find the way to express this, it would be beautiful to paint this group…”.

It is up to you to decide whether or not Rivera succeeded. In any case, and along the same line, it is worthwhile to read this fragment from Ibn Al-Nafis’ “Commentary on Anatomy in Avicenna’s Canon” as quoted in an article dedicated to the history of pulmonary circulation by El Maghawry, Zanatta, and Zampieri [3]:

“When hearing something unusual, do not preemptively reject it, for that would be folly. Indeed, unconventional things may be true, and familiar and praised things may prove to be lies. Truth is truth unto itself, not because many people say it is.”
